# Relaxin in fibrotic ligament diseases: Its regulatory role and mechanism

**DOI:** 10.3389/fcell.2023.1131481

**Published:** 2023-04-11

**Authors:** Shuai Yuan, Dong Guo, Xinzhi Liang, Luhui Zhang, Qun Zhang, Denghui Xie

**Affiliations:** ^1^ Department of Joint Surgery and Sports Medicine, Center for Orthopedic Surgery, Orthopedic Hospital of Guangdong Province, The Third Affiliated Hospital of Southern Medical University, Guangzhou, China; ^2^ Guangdong Provincial Key Laboratory of Bone and Joint Degeneration Diseases, Academy of Orthopedics, Guangdong Province, Guangzhou, Guangdong, China; ^3^ Good Clinical Practice Development, Guangdong Provincial Key Laboratory of Bone and Joint Degeneration Diseases, The Third Affiliated Hospital of Southern Medical University, Guangzhou, China

**Keywords:** relaxin, fibrosis, ligament, TGF-β, fibroblast

## Abstract

Fibrotic ligament diseases (FLDs) are diseases caused by the pathological accumulation of periarticular fibrotic tissue, leading to functional disability around joint and poor life quality. Relaxin (RLX) has been reported to be involved in the development of fibrotic lung and liver diseases. Previous studies have shown that RLX can block pro-fibrotic process by reducing the excess extracellular matrix (ECM) formation and accelerating collagen degradation *in vitro* and *in vivo*. Recent studies have shown that RLX can attenuate connective tissue fibrosis by suppressing TGF-β/Smads signaling pathways to inhibit the activation of myofibroblasts. However, the specific roles and mechanisms of RLX in FLDs remain unclear. Therefore, in this review, we confirmed the protective effect of RLX in FLDs and summarized its mechanism including cells, key cytokines and signaling pathways involved. In this article, we outline the potential therapeutic role of RLX and look forward to the application of RLX in the clinical translation of FLDs.

## 1 Introduction

Fibrosis is defined as the overgrowth, hardening, and/or scarring of various tissue due to excessive deposition of extracellular matrix (ECM) components such as collagen (Kisseleva and Brenner, 2008; Henderson et al., 2020). Fibrosis is divided into four phases: 1) acute injury stage, 2) inflammatory response stage, 3) fibroblast-to-myofibroblast transformation phase, and 4) remodeling stage, including extracellular proteolysis and internal degradative endocytosis of the fibrous matrix (Giannandrea and Parks, 2014). Fibrotic ligament diseases (FLDs) are commonly seen in many countries all around the world and include adhesive capsulitis, carpal tunnel syndrome, Dupuytren’s disease, cubital tunnel syndrome, arthrofibrosis, and scleroderma. There are similar pathological changes in the above-mentioned areas: the excessive accumulation of fibrotic tissue (Bournia et al., 2009; Kang et al., 2014; Kang et al., 2017; Blessing et al., 2019; Ko et al., 2019).

So far, there are no ideas and effective methods to address the root cause of FLDs. Currently, most non-surgical treatments, including physical therapy, oral anti-inflammatory drugs, and topical steroid injections, fail to reduce collagen production and accelerate collagen degradation to reverse the progression of fibrosis in the long term. Surgical treatment could eliminate the scarring but did not prevent the risk of recurrence (Neviaser and Hannafin, 2010; Padua et al., 2016; Ruettermann et al., 2021; Zhao et al., 2022a). Recently, multiple studies had reported that relaxin had the potential to inhibit fibrosis formation and reduce the risk of developing fibrosis by regulate collagen production and degradation (Bournia et al., 2009; Kang et al., 2014; Kang et al., 2017; Blessing et al., 2019; Ko et al., 2019).

Relaxin (RLX) is a polypeptide hormone (6 kDa) and mainly produced by the ovaries, placenta during pregnancy as well as the prostate gland in mammal. RLX was first reported to act on the pubic symphysis, dilating the birth canal and facilitating fetal delivery (Hisaw, 2016). It was later found to suppress fibrosis by inhibiting collagen production and promoting collagen degradation (Franklin, 1997). The RLX polypeptide family is encoded by seven genes in humans, including three RLX genes *RLN1, RLN2, and RLN3*, and four insulin-like peptide genes, *INSL3,INSL4, INSL5, and INSL6* (Samuel et al., 2017). The RLX receptor 1 (RXFP1) is a leucine-rich-repeat (LGR)-containing G protein-coupled receptor (GPCR) that mediates the most of biological processes of RLX-2. Many studies have shown that RLX-2 plays a key regulatory role in ECM remodeling (Sassoli et al., 2022).

Recombinant RLX-2 has been reported to exert anti-fibrotic effects in fibrotic diseases of heart, liver, kidney, lung, and skin (Unemori et al., 1996; Seibold et al., 2000; Dschietzig et al., 2006; Bathgate et al., 2013). Table 1 summarizes the regulation of RLX in different organs and FLDs. In recent years, RLX has been shown to reduce the ECM formation and promote ECM degradation in a rat shoulder joint immobilization model (Blessing et al., 2019; Kirsch et al., 2022)*.* However, the underlying mechanisms of RLX remain unclear, especially the major cellular, molecular and signaling pathways.

**TABLE 1:** Animal models and the effect of RLX applied to different organs.

Organs	Animal models	Effect of RLX
Heart	Myocardial ischemia model (Hirata et al. (2015); Angiotensin II mice (Wilhelmi et al. (2020)TABLE; Transverse aortic constriction model Wilhelmi et al. (2020); Isoproterenol induced mice (Cai et al. (2017)	Alleviating cardiac fibrosis Wilhelmi et al. (2020), Wu et al. (2018), Cáceres et al. (2019), Samuel et al. (2014)
Liver	CCL4 mouse model Ravichandra and Schwabe (2021); Bile duct ligation (BDL) model Ravichandra and Schwabe (2021); Diethyl nitrosamine (DEN) rat model Qu et al. (2018)	Reducing hepatic fibrosis Bennett et al. (2017)
Lung/Trachea	Bleomycin-induced mice Liu et al. (2017); Silica aerosolized model Barbarin et al. (2005); Fluorescein isothiocyanate induced model Roberts et al. (1995); Irradiation-induced pulmonary fibrosis in the mouse McDonald et al. (1993); Human fibroblasts transplantation in immunodeficient mice Phillips et al. (2004); Ovalbumin -induced chronic allergic airways disease in mice Huuskes et al. (2015)	Reducing fibrosis and related airway dysfunction Royce et al. (2019); Abrogating established airway fibrosis Royce et al. (2015)
Renal	Drug induced models Nogueira et al. (2017) (HgCl2, Vanadate, Adriamycin, Uranyl nitrate, Folic acid, *etc.*); Surgical induced models: Ureteral obstruction (UUO) model Chevalier et al. (2009); Kidney ischemia mice Takada et al. (1997)	Attenuating renal inflammation and fibrosis Giam et al. (2018), Li et al. (2021), Wetzl et al. (2016), Huuskes et al. (2015)
Skin	Radiation ulcers Zhao et al. (2019); Bleomycin-induced skin fibrosis Błyszczuk et al. (2019); Skin wound healing models Wilhelm et al. (2017); Vinyl chloride induced model Christner et al. (2000)	Attenuating skin fibrosis Corallo et al. (2019); Coentro et al. (2021)

In this review, we provide an overview of the regulatory role of RLX in various fibrotic diseases and summarize its mechanisms, especially the crucial cells, cytokines and signaling pathways involved. The effectiveness of RLX in the treatment of FLDs was further confirmed, and the potential mechanism of RLX in the treatment of FLDs was updated. Based on the review, the new insights into RLX in FLDs are systematically introduced and the potential clinical implementation of RLX as a new therapeutic target for FLDs is highlighted.

## 2 Key cell involved in fibrosis

**FIGURE 1:**
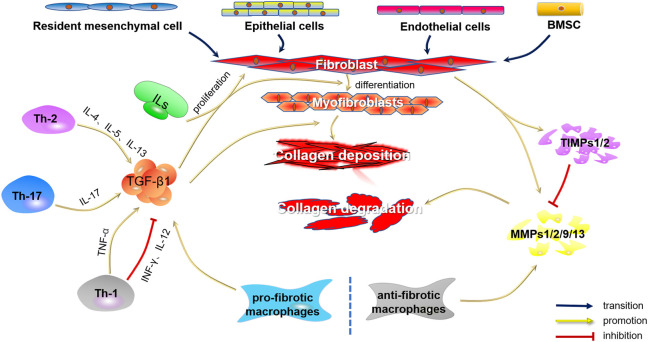
Cells and cytokines involved in fibrosis.

Fibrosis is a regulatable process in which many types of cells play vital roles. Therefore, in this article, we focus on several key cells involved in fibrosis, including fibroblasts, T cells, monocytes, and macrophages, and the secretion of critical cytokines will also be described such as interleukins, transforming growth factor-β1 (TGF-β1), matrix metalloproteinases (MMPs) and tissue inhibitor of matrix metalloproteinases (TIMPs). Figure 1 outlines the cells and cytokines involved in fibrosis.

### 2.1 Fibroblasts and myofibroblast

Fibroblasts, considered the major cells in most fibrotic diseases, were activated to secrete ECM, including fibronectin, collagen types I, III, and IV, leading to the formation of scar tissue after injury (Bucala et al., 1994). Fibroblasts could be regulated and transformed into myofibroblasts by factors they produced and various paracrine signals from lymphocytes. Meanwhile, myofibroblast-derived molecules also played a role in the transformation (Wynn, 2008). However, the source of myofibroblasts varied in different fibrotic diseases. In addition to resident fibroblasts, myofibroblasts could also originate from epithelial-mesenchymal transition (EMT) of epithelial cells, endothelial-mesenchymal transition (EndMT) of endothelial cells, or recruitment from the bone mesenchymal stromal cells (Bucala et al., 1994; Kalluri and Neilson, 2003; Quan et al., 2006; Willis et al., 2006; Zeisberg et al., 2007; Kisseleva and Brenner, 2008; El Agha et al., 2017). Although myofibroblasts are produced differently in different fibrotic diseases, the stages of ECM production by myofibroblasts are similar. First, collagen-secreting myofibroblasts are derived from resident fibroblasts, EMTs, EndMTs, or mesenchymal stromal cells, second, myofibroblasts produce procollagen molecules, which are processed and assembled for release into the extracellular matrix. The procollagen in the ECM was then modified with N-proteinases and C-proteinases to form collagen monomers. Finally, collagen monomers are polymerized into fibrils by fibronectin (Gelse et al., 2003; Canty and Kadler, 2005). Fibrosis occurred when the rate of ECM production exceeded the rate of degradation. Some findings showed that serelaxin, a recombinant form of RLX-2, could inhibit the differentiation of fibroblasts into myofibroblasts by inhibiting the ALK-5/Smad2/3 pathway and increasing the ratio of MMP-2/TIMP-2, thereby reduce collagen production (Wu et al., 2018). Since the conversion of fibroblasts to myofibroblasts played a vital role in fibrotic diseases, inhibition of this process became an important basis for RLX-2 to alleviate fibrosis.

### 2.2 Th-2 cells and eosinophils

Among factors that induce fibrosis, cytokines secreted by Th-2 cells were first recognized to have potent profibrotic properties. The classic cytokines released by Th-2 cells were IL-4, IL-5, IL-10, and IL-13. Previous studies had reported that IL-4, IL-5, and IL-13 were associated with the development of fibrosis (Mack, 2018). IL-4 is a crucial fibrosis-promoting cytokine that exerts pro-fibrotic activity by increasing the synthesis of collagen and other matrix proteins (Fertin et al., 1991; Postlethwaite et al., 1992). However, recent studies have shown that IL-4 has a dual role in promoting fibrosis and resisting fibrosis (Izbicki et al., 2002; Huaux et al., 2003; Liang et al., 2017; Zhang et al., 2017). IL-5 had been shown to regulate the proliferation, mobilization, and activation of eosinophils, and activated eosinophils could produce cytokines that promote fibrosis. Gharaee-Kermani et al. (1998) had reported that treatment with anti-IL-5 antibodies could reduce pulmonary eosinophilia, cytokine expression, and fibrosis in bleomycin-induced pulmonary fibrosis in mice. Furthermore, injection of anti-IL-5 monoclonal antibody or the use of IL-5-deficient mice as recipients also resulted in the lack of eosinophil infiltration or dermal fibrosis in chronic skin allograft rejection (Le Moine et al., 1999). Indeed, IL-5 could exert a pro-fibrotic effect by mediating the expression of IL-13 in eosinophils (Reiman et al., 2006). At the same time, some studies had shown that IL-13 could inhibit the production of matrix metalloproteinase-1 (MMP-1) and MMP-3, increase the production of tissue inhibitor of metalloproteinase-1 (TIMP-1), and play a role in promoting the production of collagen (Oriente et al., 2000). The profibrotic effects of IL-13 were mediated by two IL-13 receptors. On the one hand, IL-13 induced macrophages to upregulate IL-13Ra2, and macrophages bind IL-13 to release pro-fibrotic TGF-β1 (Fichtner-Feigl et al., 2006). On the other hand, IL-13Ra2 had also been shown to have anti-fibrotic properties. The results showed that overexpression of IL-13Rα2 in mice lung attenuated bleomycin-induced lung fibrosis (Lumsden et al., 2015). Although the role of IL-4, IL-5, and IL-13 have been elucidated in several diseases, it was unclear whether they have similar regulatory effects on fibrosis in FLDs. On the other hand, in order to determine the anti-fibrotic mechanism of RLX-2, it would be worth investigating whether RLX-2 plays an anti-fibrotic role by regulating the secretion of IL-4 and IL-5 by Th-2 cells and the expression of IL-13 by eosinophils.

### 2.3 Th-17 and Th-1 cells

T-helper 17 (Th17) cells belonged to the CD41^+^ T cell lineage and were characterized by the production of interleukin 17A (IL-17A), a founding member of the IL-17 cytokine family and a characteristic cytokine of theTh-17 cell population (Fouser et al., 2008; Akbar et al., 2021a). Meanwhile, IL-17A was a cytokine that mediated inflammation (Shen et al., 2009), fibrosis (Tan et al., 2013), and pain signaling (Sun et al., 2017). Many recent reports suggested that IL-17 had direct and indirect pro-fibrotic properties. For example, IL-17 could play a pro-fibrotic role in liver (Tan et al., 2013), lung (Wang et al., 2017), renal (Peng et al., 2015) and heart (Li et al., 2014). Fibroblasts have been found to be among the most sensitive cells to IL-17A in many fibrotic diseases. Some gene expression data showed that the expression of IL-17A was significantly increased in FLDs, and IL-17A increased the sensitivity of fibroblasts in frozen shoulder tissue. Meanwhile, IL-17A could upregulate the gene expression of COL3A1, MMP-1, and MMP-3 by inducing mitogen-activated protein kinase, nuclear factor κB (NF-κB), phosphoinositide 3-kinase (PI3K), and C/EBP signaling pathways in frozen shoulder fibroblasts. Therefore inhibition of IL-17A signaling might be a viable approach to target fibrosis and inflammation in frozen shoulder (Akbar et al., 2021a). In fact, IL-17A and RLX share the same downstream signaling pathway in fibrotic diseases, but whether RLX exerts its antifibrotic effect by regulating IL-17A levels in fibroblasts or whether RLX and IL-17A have a crosstalk effect on fibrosis remains to be proved experimentally.

In addition to Th-17 cells, Th-1 cells had also been found to promote fibrosis. Th-1 cells were characterized by the production of IFN-γ, IL-2, IL-6, IL-12, IL-21, and TNF-α (Ranieri et al., 2021). Studies had shown that the classical Th-1 cytokine IFN-γ (Baroni et al., 1996; Oldroyd et al., 1999) and the Th-1-inducing cytokine IL-12 (Wynn et al., 1995; Keane et al., 2001) could attenuate fibrosis in lung, liver and kidney fibrosis models by antagonizing the activity of TGF-β. In contrast to IFN-γ, the Th-1 cytokine TNF-α exhibited pro-fibrotic properties in various animal models. The results showed that administration of TNF-alpha blockers or TNF-alpha receptor-deficient mice reduced fibrosis in organs such as the kidney and liver (Therrien et al., 2012; Wen et al., 2019; Zhang et al., 2019; Liang et al., 2021). In fact, IL-6 was required for Th17 cells differentiation (Peng et al., 2015). Studies had shown that IL-6 has pro-fibrotic activity in lung (Ayaub et al., 2017), heart (Kumar et al., 2019), kidney (Chen et al., 2019), liver (Xiang et al., 2018), and other organ models of fibrosis. Other reports also confirmed that IL-6 has a pro-fibrotic effect by regulating the TGF-β pathway (O'Reilly et al., 2014; Zhang et al., 2012). Beiert et al. found that the pro-fibrotic effect of IL-6 was regulated by RLX, which reduced the formation of fibrotic tissue by reducing the level of IL-6 transcripts in the mouse heart (Beiert et al., 2018). The finding would be a powerful force in the evidence that RLX attenuates fibrosis.

In summary, recent studies have shown that Th17 and Th-1 cells can induce fibrosis, so lymphocyte may play a vital role in fibrosis and inhibiting lymphocyte activity may become a new way to inhibit fibrosis. Meanwhile, in order to further elucidate the anti-fibrosis activity of RLX, we speculate that RLX may exert its anti-fibrosis effect by acting on Th-17 and/or Th-1 cells.

### 2.4 Macrophages

Macrophages normally had dynamic homeostatic functions, such as clearance of tissue debris and apoptotic cells, suppression of tissue inflammatory responses, provision of initial defense against microbial threats, and promotion of ECM turnover. An altered homeostatic microenvironment facilitated the recruitment of macrophages from monocytes to defend against threats and promote wound healing after tissue injury (Zhao et al., 2022b). To promote fibrosis, monocyte-derived macrophages produced a variety of factors that affect fibrosis and tissue regeneration, mainly transforming growth factor-β1 (TGF-β1), platelet-derived growth factor (PDGF), MMPs. (Mack, 2018). Macrophage-derived TGF-β could promotes fibroblast proliferation, activation, and collagen synthesis (Fine and Goldstein, 1987; Clark et al., 1997; Acharya et al., 2008). Studies have shown that macrophages played a crucial role in the process of liver fibrosis. Macrophages activated hepatic stellate cells by producing profibrotic factors such as TGF-β and PDGF. Activated hepatic stellate cells (aHSCs), the main effector cells of liver fibrosis, could be reversed into the quiescence *in vivo*. Recent studies had shown that RLX-2, as an endogenous peptide hormone, played a key role in anti-hepatic fibrosis. Hu et al. had found that RLX-treated macrophage-derived exosomes could convert aHSCs to a quiescent state by upregulating miR-30a-5p, thereby exerting anti-fibrotic effects (Hu et al., 2021).

In fact, macrophages were intricately involved in the regulation of fibrosis. Monocyte-derived macrophages could be polarized in several directions. IFN-γ, LPS and TNF-α could promote M1 polarization, while IL-4, IL-13, and IL-10 could promote M2 polarization. M1 polarized macrophages predominantly expressed inducible nitric oxide synthase (iNOS), high levels of IL-12, and other pro-inflammatory cytokines such as IL-1, IL-6, and TNF-α. M2 polarized macrophages express arginase, matrix proteins, and cytokines like IL-10, TGF-β, and IL-1 receptor antagonist (Murray, 2017). However, the original concept of two distinct phenotypes of macrophages, M1 and M2, had been challenged to adequately explain the mechanisms by which macrophages were involved in fibrosis. To further elucidate the role of macrophages in the process of fibrosis, several studies had divided macrophages into pro-fibrotic macrophages and anti-fibrotic macrophages. Pro-fibrotic macrophages promoted collagen deposition by secreting fibroblast factors (TGF-β, IL1, IL-6, IL-12, *etc.*), while anti-fibrotic macrophages could not only degrade collagen by secreting a variety of matrix degrading enzymes, but also degrade collagen through the lysosomal pathway (Adhyatmika et al., 2015; Zhao et al., 2022b). However, the surface markers of pro-fibrotic macrophages and anti-fibrotic macrophages remain unclear. Therefore, effectively distinguishing pro-fibrotic macrophages from anti-fibrotic macrophages will help to fully elucidate the regulatory role of macrophages in the process of fibrosis.

In conclusion, macrophages were a double-edged sword in fibrosis. On the one hand, macrophages appear to promote fibrosis, and on the other hand, they appear to play a crucial role in anti-fibrosis. In the early stages of FLDs, macrophages appear to be the main cells that can promote fibroblast differentiation by releasing various factors. Therefore, inhibiting macrophage activity may be an effective way to inhibit the progression of fibrosis. In fact, macrophages expressed abundant RXFP1 (Horton et al., 2011). RLX-2 may promote anti-fibrotic macrophage proliferation to inhibit fibrosis through RXFP1. Therefore, understanding the mechanism of how RLX-2 stimulates macrophages differentiation *in vivo* is essential to fully elucidate the anti-fibrotic activity of RLX-2.

## 3 Key cytokines involved in fibrosis

Fibrosis was known as a pathological process of disease regulated by several key cytokines, including transforming growth factor-β1 (TGF-β1), matrix metalloproteinases (MMPs), tissue inhibitors of matrix metalloproteinases (TIMPs) and vascular endothelial growth factor (VEGF). To better elucidate this process, it is necessary to analyze some important cytokines.

### 3.1 TGF-β1

TGF-β was cytokine family, which consists of three members (TGF-β1, β2, and β3). Of the three forms of TGF-β, TGF-β1 had been shown to be a master regulator of fibrosis (Meng et al., 2016). According to the previous studies on FLDs, TGF-β1 could promote the production and deposition of the extracellular matrix (ECM) through the TGF-β1/Smad-3 signaling pathway (Kim et al., 2018). The ECM represented a complex of protein families, the most common of which was the collagen family (Gelse et al., 2003), especially types I and III predominate in fibrosis (Kisseleva and Brenner, 2008). Studies showed that the expression of the COL1A2 gene was regulated by TGF-β1 (Zhang et al., 1998; Ghosh et al., 2000). Collagen types I and III were expressed to restore tensile strength and tissue integrity, and TGF-β1 was an inducible cytokine required for the production of these collagens after tissue injury during injury repair (Kim et al., 2018). In fact, TGF-β1 was not only involved in the transcription of type I collagen, but also could be involved in the translation of type I collagen. It was found that TGF-β1 participated in the expression, secretion, and deposition of collagen in the ECM by regulating of miRNAs (Yang et al., 2013; Das et al., 2014). And heat shock protein 47 (HSP47) and FK506-binding protein 10 (FKBP10) also played a regulatory role in procollagen assembly and transport. These proteins prevented procollagen degradation and premature formation during procollagen formation and transport, however, the expression of HSP47 and FKBP10 was regulated by TGF-β1 in fibroblasts (Bellaye et al., 2014; Staab-Weijnitz et al., 2015; Ito and Nagata, 2017). In addition to promoting the collagen assembly, TGF-β1 also induced the expression of protease inhibitors, such as plasminogen activator inhibitor 1 (PAI-1) and TIMP-3, which could attenuate the degradation of deposited collagen by proteases (Rabieian et al., 2018; de Oliveira and Wilson, 2020). In summary, it had been established that TGF-β1 was involved in the expression of collagen genes, as well as collagen transport, deposition, and degradation. So, inhibiting the expression of TGF-β1 could likely be an effective intervention in anti-fibrosis. Interestingly, serelaxin, a recombinant relaxin-2, had been proved to suppress the TGF-β1/IL-1 axis to inhibit myofibroblast differentiation and collagen deposition by targeting TLR-4 and NLRP3 inflammatory bodies in cardiac myofibroblasts (Cáceres et al., 2019). However, how RLX-2 alleviates fibrosis by regulating the TGF-β signaling pathway in FLDs remains unclear. Therefore, a large amount of experimental evidence is still needed.

### 3.2 MMPs and TIMPs

Matrix metalloproteinases (MMPs), belonging to a multigene family secreted by connective tissue cells and inflammatory phagocytes, played a key role in ECM remodeling due to their ability to degrade many matrix components, growth factors and cytokines (Nagase and Woessner, 1999). Under physiological conditions, the proteolytic activity of MMPs was tightly controlled by their endogenous protein inhibitors (TIMPs) (Gomez et al., 1997). During fibrosis, MMPs and TIMPs were unbalanced, MMPs activity was inhibited and TIMPs expression was increased, ECM was protected, and collagen degradation was reduced. Myofibroblasts at the site of injury were driven by inflammatory factors to synthesize new collagen during fibrosis. When the rate of collagen synthesis exceeded the rate of degradation, abnormal collagen would be deposited in ECM, further leading to fibrosis and pathological remodeling of tissue and organs (Van Linthout et al., 2014). Indeed, the biological roles of MMPs in fibrosis were not fully established, but they appeared to vary with the particular family member, the tissue involved and the stage of the fibrotic response. Notably, some members of this family exhibited pro-fibrosis effects, while others act as anti-fibrotic molecules. For example, MMP-13 expression levels were elevated in early stages of liver fibrosis models (Watanabe et al., 2000). Notably, high expression level of MMP-13 resulted in upregulation of pro-fibrotic cytokines such as IL-1α, IL1β and TNF-α, suggesting that the matrix metalloproteinase family was not only involved in collagen degradation but also in the process of collagen formation. Studies described the interaction of MMPs and TIMPs in fibrosis. The results showed that MMP-2 was activated after the interaction of MMP-14 and TIMP-2 (Bassiouni et al., 2021) and activated MMP-2 could inhibit cardiac fibrosis by inhibiting TIMP-1 (Onozuka et al., 2011). Silicosis was a common condition associated with pulmonary fibrosis. Some results indicated that RLX could improve silica-induced pulmonary fibrosis by increasing MMP-2 expression (Li et al., 2013). In addition to MMP-2, MMP-1 was also involved in fibrosis. In a thioacetamide-induced liver fibrosis model, after infection of rats with recombinant adenovirus, Ad5MMP-1 (human pro-human matrix metalloproteinase-1 complementary DNA), rat liver fibrosis was attenuated and the high expression level of MMP-1 increased hepatocyte proliferation while also caused an appropriate amount of damage to other tissue (Iimuro et al., 2003). In the experiment studying the effect of RLX on the cervix, Gerson Weiss found that RLX could significantly increase the expression levels of MMP-1 and MMP-3, and significantly inhibit the expression of its endogenous inhibitor TIMP-1 to prepare for labor (Weiss and Goldsmith, 2005). In addition to MMP-1 and MMP-2, studies had also shown that MMP-8 had an anti-fibrotic effect. Similar to studies involving MMP-1, MMP-8-carrying adenovirus induced the degradation of type I and type III matrix collagens in mice by increasing the expression of MMP-2 and MMP-3 to reduce liver fibrosis (Siller-López et al., 2004). RLX could also affect the expression of MMP-8. Studied showed that RLX could significantly increase the expression of MMP-1 and MMP-8 in human periodontal ligament (hPDL) cells *in vitro* over time, further regulating collagen metabolism (Hirate et al., 2012).

Four distinct members have been identified in mammals: TIMP-1, TIMP-2, TIMP-3, and TIMP-4 (Hemmann et al., 2007). TIMPs, as an endogenous inhibitor of MMPs, also played a crucial role in the occurrence and development of fibrosis. TIMPs varied in different fibrotic diseases. In a mouse model of liver fibrosis, H Yoshiji found that TIMP-1 overexpression resulted in more severe fibrosis in a mouse liver fibrosis model, but had no significant effect on collagen synthesis (Yoshiji et al., 2000), whereas an increase in fibrosis was observed in mice whose TIMP-1 gene had been knocked out (Timp-1^−/−^ mice) (Wang et al., 2011). However, results from mouse models of unilateral ureteral obstruction (UUO) and protein overload-induced renal fibrosis showed that TIMP-1 deficiency had no effect on disease severity (Eddy et al., 2000; Kim et al., 2001). Therefore, TIMP-1 might not be a determinant for promoting liver fibrosis. In addition to TIMP-1, TIMP-2 had also been shown to be a potent pro-fibrotic factor. In a CCL4-induced liver fibrosis model, TIMP-2 siRNA knockdown mice exhibited suppression of hepatic stellate cells and reduced collagen deposition, suggesting a pro-fibrotic role for TIMP-2 (Hu et al., 2007). RLX had the effect of decomposing collagen, which had been confirmed in previous studies and reports studies. Some studies had also clarified that the anti-fibrosis effect of RLX was related to TIMP-1 and TIMP-2. Williams et al. (2001) found that RLX mediated the reduction of collagen deposition not only directly by reducing type I collagen synthesis, but also indirectly by reducing the expression of TIMP-1 and TIMP-2. Similar to TIMP-1 and TIMP-2, the effect of TIMP-3 in fibrotic diseases deserved attention. In TIMP-3^−/−^ mice with bleomycin-induced pulmonary fibrosis, the level lung fibrosis was increased and persisted, and it was also found that despite an overall increase in metalloproteinase activity, TIMP-3^−/−^ mice Pulmonary fibrosis was enhanced (Gill et al., 2010), suggesting that the function of MMPs could not be understood merely as the degradation or removal of ECM. Like pulmonary fibrosis, kidney fibrosis was enhanced in TIMP-3^−/−^ mice had aggravated renal fibrosis after 2 weeks of unilateral ureteral obstruction (UUO), and Kassiri suggested that the protective effect of TIMP-3 was due to inhibition of TNF-α-mediated renal fibrosis and regulation of expression of multiple MMPs (Kassiri et al., 2009). This study contrasted with a previous study in which Kawamoto found no difference in renal fibrosis between wild-type and TIMP-3^−/−^ mice after 7 days of UUO treatment. However, they detect increased metalloproteinase activity and turnover of TGF-β1 in TIMP-3^−/−^ mice (Kawamoto et al., 2006). Some reports had shown that Dupuytren myofibroblasts treated with adenoviral RLX construct showed increased TIMP-3 protein expression. These results suggested that TIMP-3 might play a protective role in fibrotic diseases.

In fact, in the existing RLX studies of FLDs, there are certain differences in the expression of MMPs and TIMPs in diseases, which may explain that fibrosis is a dynamic pathological process involving many cells and cytokines. However, the regulatory roles of RLX on MMPs and TIMPs remain unclear. We speculate that RLX plays an anti-fibrotic role by promoting the expression of MMPs and downregulating the expression of TIMPs, but which MMPs and TIMPs play crucial roles in anti-fibrosis remains to be determined. Therefore, elucidating the specific role of MMPs and TIMPs in collagen degradation can not only explain the anti-fibrotic activity of RLX, but also provide some reference indicators for other antifibrotic studies.

### 3.3 VEGF

Previous studies on fibrosis in liver (Yang et al., 2014), lung (Barratt et al., 2017) and other organs have shown that inhibiting vascular endothelial growth factor (VEGF) expression could alleviate fibrosis. Recently, studies had reported that VEGF might play a vital role in anti-fibrosis (Chellini et al., 2018). At the same time, studies had shown that RLX could induce endometrial stromal cells, cardiac fibroblasts, and THP-1 monocytes to produce VEGF (Unemori et al., 2000; Palejwala et al., 2002; Formigli et al., 2007; Sarwar et al., 2015) and induce angiogenesis, thereby enhancing tissue perfusion, especially ischemia-reperfusion organs to promote wound healing. Although the property of VEGF had been shown to inhibit the TGF-β -1 mediated epithelial-mesenchymal transition by inhibiting myofibroblast differentiation (Hong et al., 2013; Chellini et al., 2018), more experiments were needed to determine whether VEGF played an anti-fibrotic role in FLDs and whether anti-fibrotic activity of RLX was partially induced by VEGF. In some experiments, we found that TGF-β-Smad2/3 signaling pathway can mediate the expression of VEGF to promote angiogenesis (Xi et al., 2021). Abnormal angiogenesis is the early pathological manifestation of FLDs (Zhao et al., 2021). We speculate that the anti-fibrotic effect of RLX may be mediated by inhibiting the TGF-β-Smad2/3 signaling pathway, which inhibits the production of VEGF and then exerts anti-fibrotic effects in the early stage of the disease. However, reliable experimental evidence is still needed.

## 4 Key anti-fibrotic signaling pathways of RLX

**FIGURE 2:**
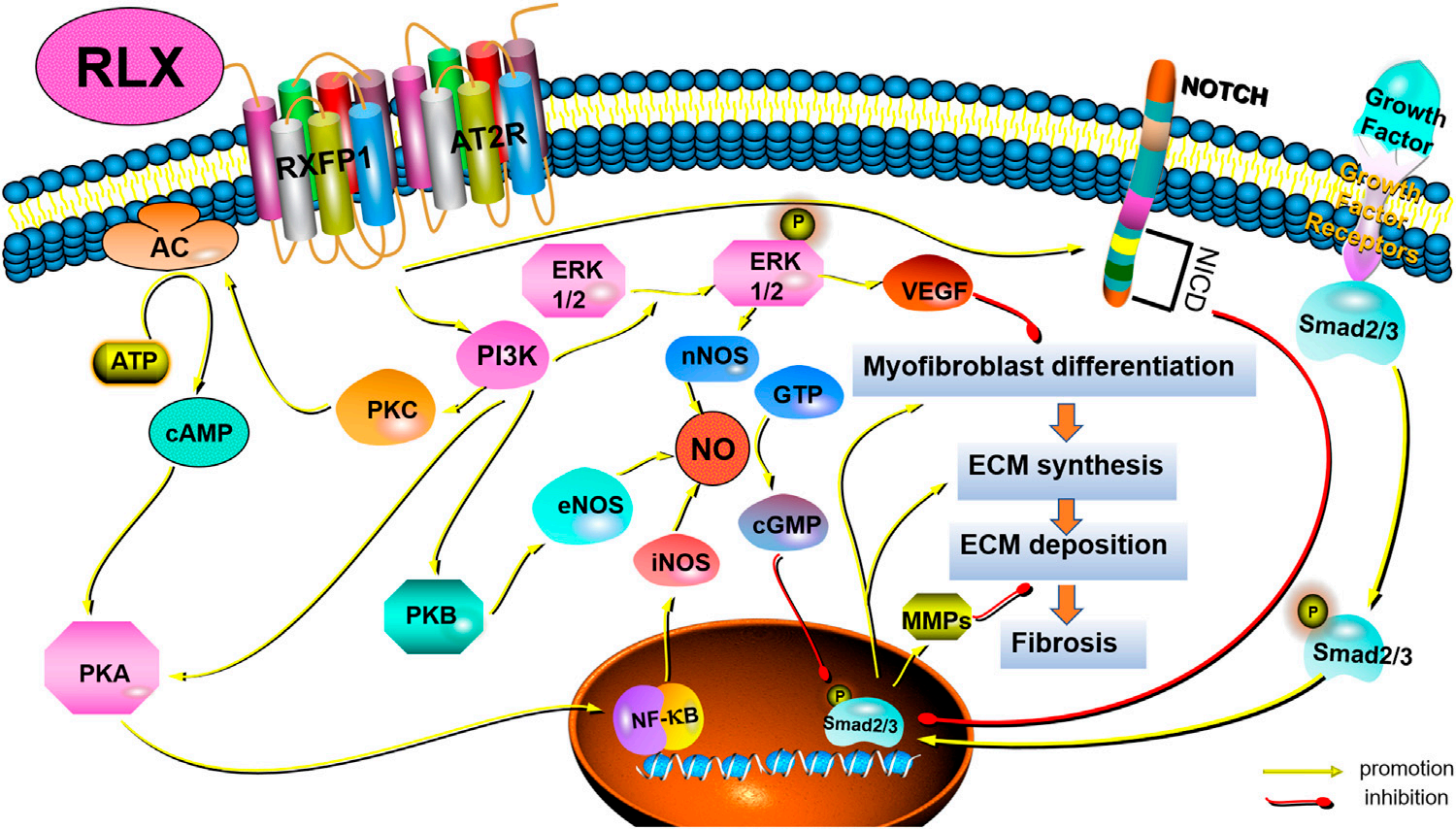
Schematic drawing of the main anti-fibrotic signaling transduction mechanisms of RLX/RXFP1.

Pathological tissue remodeling was considered a hallmark of fibrosis (Eming et al., 2017). The molecular mechanisms leading to fibrosis were complex. Fibrosis was a dynamic process with strong plasticity, and a variety of signaling pathways participate in the regulation of the occurrence and development of fibrosis. However, the anti-fibrotic mechanism of RLX in FLDs remains unclear. To fully elucidate the potential signaling pathways of RLX in FLDs, we analyzed signaling pathways known to suppress organ fibrosis. Table 2 summarizes the main signaling pathways and roles of RLX in fibrosis. RLX appears to inhibit the myofibroblast differentiation, activate MMPs, and neutralize the effects of TGF-β1 to suppress fibrosis through these pathways (as shown in Figure 2).

**TABLE 2:** Signaling pathways and effect involved in RLX.

Cells	Key signaling pathways	Effect of RLX
Human coronary artery endothelial cells (HCAECs) and mouse cardiac endothelial cells (MCECs) Wilhelmi et al. (2020); Renal myofibroblasts Meng et al. (2016) Human endometrial stromal cells Zhang et al. (2002); Human umbilical vein endothelial, epithelial, and vascular smooth muscle cells Dschietzig et al. (2003); H9c2 cardiomyocytes Boccalini et al. (2018)	TGF-β1 -pSmad2/3 Meng et al. (2016); Wilhelmi et al. (2020); TGF-β1-MAPK-ERK1/2 Zhang et al. (2002); TGF-β1-PI3K Sánchez et al. (2018); Valkovic et al. (2019)	Angiogenesis Zhang et al. (2002); Vasodilation Zhang et al. (2002); Valkovic et al. (2019); Apoptosis Valkovic et al. (2019); Anti-fibrosis Meng et al. (2016); Valkovic et al. (2019); Wilhelmi et al. (2020)
Mast cells Masini et al. (1994); Lung fibroblasts Huang et al. (2011); Renal myofibroblasts Wang et al. (2016); Vascular endothelial cells Bani-Sacchi et al. (1995); Smooth muscle cells Bani et al. (1998)	nNOS and eNOS-NO/cGMP and iNOS - NO/cGMP Nistri and Bani (2003); ET1-32-ET-B-eNOS-NO/cGMP Conrad and Novak, (2004)	Vasodilation Bani-Sacchi et al. (1995); Bani et al. (1998); Anti-fibrosis Huang et al. (2011); Wang et al. (2016); Wetzl et al. (2016); Stimulating the production of endogenous NO Nistri and Bani (2003); Conrad and Novak (2004)
Human coronary artery endothelial cells (HCAECs) and mouse cardiac endothelial cells (MCECs) Wilhelmi et al. (2020) Mouse cardiac muscle cells and rat H9c2 cardiomyoblasts Frati et al. (2015)	Notch-1 Wilhelmi et al. (2020) Sphingosine-1-phosphate (S1P) Frati et al. (2015)	Anti-fibrosis Frati et al. (2015); Wilhelmi et al. (2020)
Myofibroblast Samuel et al. (2017); Rat kidney myofibroblasts Chow et al. (2014)	RXFP1-TGF-βR Samuel et al. (2017) RXFP1-AT2R Chow et al. (2014)	Anti-fibrosis Chow et al. (2014); Samuel et al. (2017)

### 4.1 TGF-β1 / Smads pathways

Among the many cytokines, TGF-β1 was the main factor in regulating fibrosis (Wynn, 2008). TGF-β1 could regulate fibrogenesis in the heart (Tian et al., 2021), liver (Gough et al., 2021), kidneys (Chen et al., 2018), and pulmonary (Eser and Jänne, 2018) through canonical and non-canonical pathways. The canonical pathway was Smad-dependent pathways. In renal fibrosis, TGF-β1 activated myofibroblasts, promoted ECM formation and inhibit ECM degradation through the above signaling pathways (Meng et al., 2016). Some findings showed that serelaxin could alleviate cardiac fibrosis by inhibiting the enrichment of phosphorylated Smad2/3 at the RLX receptor 1 (Rxfp1) promoter region (Wilhelmi et al., 2020). In addition to canonical signaling pathways, non-canonical pathways also played a role in fibrosis. Non-canonical pathways were Smad-independent pathways, including mitogen-activated protein kinase (MAPK), phosphatidylinositol -3- kinase (PI3K) and Rho-like GTPase (Rho). Studies had shown that RLX also exerted an anti-fibrosis effect *via* MAPKs and PI3K-mediated signaling pathways. MAPK-ERK1/2 were G protein-activated serine/threonine kinases involved in a variety of fundamental cellular processes which play a key role in signal transduction (Sánchez et al., 2018). RLX had been shown to upregulate the expression of vascular endothelial growth factor (VEGF) by activating p42/44 mitogen-activated protein (MAP) kinase and MAPK (or ERK) kinase (MEK) (Zhang et al., 2002), thereby further inducing angiogenesis and activating endothelin-B (ET-B) receptors, causing vasodilation. In fact, RLX indirectly exerted anti-fibrotic effects by stimulating microvascular dilation (Dschietzig et al., 2003). The PI3K/Akt signaling pathway was also a typical G protein-dependent pathway, which was involved in the regulation of cell survival, proliferation and apoptosis. Activation of RXFP1 had been shown to activate PI3K/Akt signaling pathway in THP-1 monocytes, rat endothelial cells, mouse fibrochondrocytes, and H9c2 rat embryonic myocardial precursor cells (Boccalini et al., 2018). RLX could exhibit vasodilatory, anti-apoptotic and anti-fibrotic effects through the PI3K/Akt pathway (Valkovic et al., 2019). In fact, Rho GTPases were also involved in the pathogenesis of fibrosis. Findings showed that GTP-bound active Rho could interact with downstream effector proteins, such as the Rho-associated coiled-coil protein kinase (ROCK) and mouse hyaline-associated formin-1 (mDia1), to initiate and stabilize actin. Activation of Rho had been shown to lead to the formation of F-actin stress fiber and reduce the abundance of G-actin monomers to expose the nuclear localization sequence of myocardia-related transcription factor (MRTF). MRTF could accumulate in the nuclear and cooperate with serum response factor (SRF) to induce and maintain myofibroblasts activation, thereby promoting massive collagen production (Tsou et al., 2014). However, it remains to be seen whether RLX can act as an anti-fibrotic factor by regulating Rho GTPases.

A large number of studies have shown that RLX may play an anti-fibrotic role by regulating the TGF-β1/Smads signaling pathway. However, it is unclear how RLX regulates collagen production by inhibiting TGF-β1/Smads signaling, which may be due to direct downregulation of the expression of pro-fibrotic factors by inhibiting TGF-β1/Smads signaling in FLDs. Therefore, the impact of the interaction between the RLX and TGF-β1/Smads signaling impacts on FLDs warrants further investigation.

### 4.2 Nitric oxide synthases/NO/cGMP

The effect of RLX on NO had been demonstrated experimentally, and the results showed that RLX could exert a vasodilator effect on vascular endothelial cells and smooth muscle cells *via* the NO/cGMP system (Bani-Sacchi et al., 1995; Bani et al., 1998). Subsequent studies *in vitro* and *in vivo* revealed that RLX played a key role in anti-fibrosis *via* the NO/cGMP systems (Chow et al., 2012; Fallowfield et al., 2014; Wang et al., 2016; Bani, 2020). Nistri and Bani found that RLX could induce the activation of NO synthases, including neuronal nitric oxide synthase (nNOS) and endothelial nitric oxide synthase (eNOS), through PI3K/AKT signal pathway, and that RLX could promote the synthesis of inducible nitric oxide synthase (iNOS) *via* cAMP/PKA and/or ERK1/2 (Nistri and Bani, 2003). In addition to directly promoting the NO/cGMP system, studies also showed that RLX could converted large endothelin (ET)-1 to biologically active ET1-32 by inducing MMPs activation, binding to ET-B receptors to induce eNOS activation, which activated the NO/cGMP system on turn (Conrad and Novak, 2004). In fact, RLX appeared to indirectly exert an anti-fibrosis effect by stimulating the production of endogenous nitric oxide (NO) to stimulate microvascular dilation. Some findings showed that RLX could activate the NO/cGMP signaling pathway in lung fibroblasts and attenuated lung fibrosis (Huang et al., 2011). Meanwhile, RLX played an anti-fibrotic role in kidney myofibroblasts through activating the NO-cGMP-dependent pathway (Wang et al., 2016; Wetzl et al., 2016). Although fibroblasts and myofibroblasts are the main cells involved in fibrosis, vascular endothelial cells are also involved. We suspect that RLX exerts anti-fibrotic effects not only directly on myofibroblasts but also indirectly by affecting the microenvironment at the site of fibrosis such as tissue perfusion. However, this hypothesis requires experiments to prove.

### 4.3 Notch-1 and sphingosine-1-phosphate (S1P)

In recent years, increasing evidence had shown that RLX could inhibit connective tissue fibrosis by activating Notch-1 and sphingosine kinase/S1P signaling pathways (Sassoli et al., 2013; Frati et al., 2015). TGF-β1 induces fibrosis by downregulating Notch-1 signaling by activating transient receptor potential-canon channel (TRPC) ion channels (Sassoli et al., 2016) and voltage-gated gap junctions (Squecco et al., 2020). Wilhelmi et al. (2020) had demonstrated that serelaxin could inhibit TGFβ1-induced endothelial-mesenchymal transition by preserving Notch signaling in endothelial cells in myocardial fibrosis. Meanwhile, RLX-2 has been shown to increase sphingosine kinase activity and S1P expression and attenuate cardiac fibrosis by accelerating the secretion of MMP-2 and MMP-9 (Frati et al., 2015). Although these results suggest that RLX may exert anti-fibrotic effects through the activation of cardiac Notch-1 and sphingosine kinase/S1P pathways, the mechanism of action of these signaling pathways in FLDs remains unclear.

### 4.4 Crosstalk with other receptors and signaling pathways

In addition to directly relying on the function of RXFP1 and its signal transduction mechanism, RLX appeared to inhibit fibrosis through crosstalk with other receptors or other signaling pathways, mainly TGF-β1 receptor and the angiotensin II type 2 receptor (AT2R). Samuel et al. (2017) found that RLX could interfere with the phosphorylation of Smad2/3 and inhibit TGF-β1/TGF-βR signaling to reduce myofibroblast activation and ECM deposition. Besides, Dr. Byrna Chow and others demonstrated that AT2R antagonists could significantly block the anti-fibrotic effects of RLX *in vitro* and *in vivo*. When RLX was applied to AT2R knockout mice, it was shown that the anti-fibrotic effect of RLX requires activation of the RXFP1-AT2R heterodimer (Chow et al., 2014). The above results may provide a new direction for studying the anti-fibrotic effect of RLX in FLDs. However, applying RLX to FLDs requires the study of crosstalk between RLX and other receptors and signaling pathways. On the one hand, this will help prevent the weakening of the anti-fibrotic effect of RLX when it is used in combination with other drugs, and on the other hand, it will help to fully elucidate the pharmacological effects of RLX.

## 5 Regulation and mechanism of RLX in fibrotic ligament diseases

**FIGURE 3:**
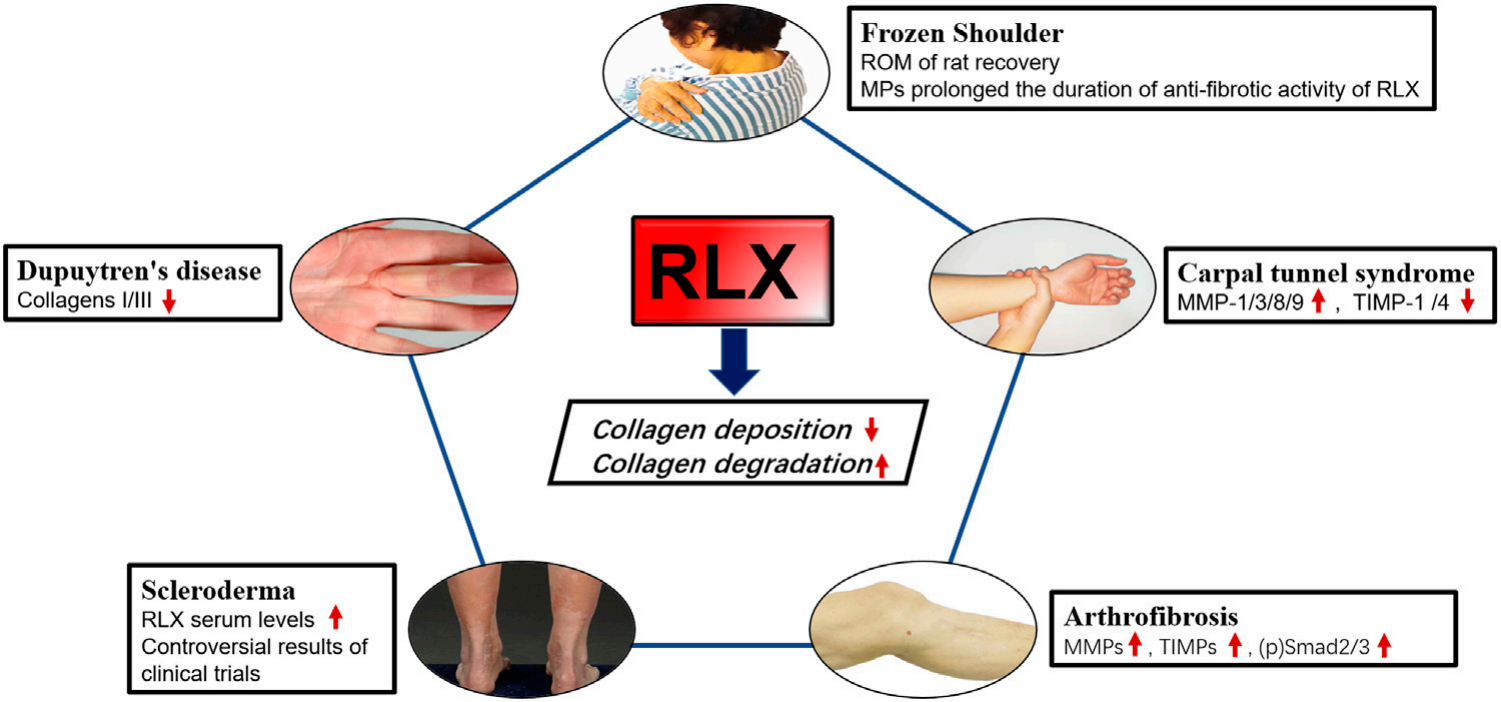
The effects of RLX in fibrotic diseases of the ligaments. Image sources of Dupuytren’s disease (Karbowiak et al., 2021) and Scleroderma (Orteu et al., 2020) are from references.

The pathological process of fibrotic ligament diseases (FLDs) is similar to other fibrous organ diseases, all of which are abnormal accumulation of collagen. RLX, as an endogenous peptide hormone, originally found to lengthen the pubic ligament, soften the organs of the birth canal and prepare for labor through the process of collagen breakdown in the pubic symphysis (Hisaw, 2016). Due to the destructive nature of collagen, several studies have been performed to investigate the effect of RLX in FLDs. Current evidence suggests that RLX attenuates fibrosis by inhibiting the recruitment and activation of myofibroblasts and reducing the expression of cytokines such as TGF-β1 (Samuel et al., 2004; Heeg et al., 2005; Mookerjee et al., 2009; Bennett et al., 2014), IL-1β (Unemori and Amento, 1990; Pini et al., 2016; Beiert et al., 2017) and TNF-α (Brecht et al., 2011; Yoshida et al., 2013), ect. In addition, RLX alleviates the fibrotic process by affecting certain signaling pathways such as TGF-β1-Smad2/3 signaling pathway. Based on the mentioned above findings, the authors analyzed the existing studies on the application of RLX in FLDs. Table 3 summarizes the human tissue sample obtaining methods and animal models for FLDs. At the same time, we investigated the role of RLX in fibrotic ligament diseases in Figure 3.

**TABLE 3:** Obtaining human Tissue samples and animal models utilized in the study of FLDs.

Diseases	Human tissue samples	Surgical/Drug-induced animal/tissue models
Frozen shoulder	Patients with primary frozen shoulder undergoing surgical arthroscopic capsular release Akbar et al. (2021b)	Rats immobilized shoulder by molding plaster Cho et al. (2019)
		Rats immobilized the humerus to the scapula Oki et al. (2015); Blessing et al. (2019)
		Rats model by injecting adenovirus-TGF-β1 into rats’ shoulder capsule Chen et al. (2021)
Dupuytren’s disease	Dupuytren’s nodule from the involutional stage during partial fasciectomy Kang et al. (2014)	Dupuytren’s disease fibroblasts transplanted to the forepaw of the athymic rat Satish et al. (2015)
		Dupuytren’s tissue samples transplanted onto chick embryo chorioallantoic membrane Mîndrilă et al. (2014)
Carpal tunnel syndrome	SSCT harvested during open carpal tunnel release Kang et al. (2017)	Hypertonic dextrose injections on the subsynovial connective tissue of rabbit Yoshii et al. (2014)
		Non-human primate model of carpal tunnel syndrome Sommerich et al. (2007)
		Altering carpal tunnel pressure for carpal tunnel syndrome of rabbit model Diao et al. (2005)
		Cutting the flexor digitorum superficialis tendon of rabbit Chikenji et al. (2014)
		Performance of a high-repetition, high-force task induces carpal tunnel syndrome in rats Clark et al. (2004)
Scleroderma	Biopsies on mid-forearm Giordano et al. (2012); Corallo et al. (2019)	Hypochlorous Acid Induced Mouse Model Meng et al. (2019)
		Xenotransplant mouse model of scleroderma Ross et al. (2021)
		Animal Models of Scleroderma: Current State and Recent Development Asano and Sato (2013)
Arthrofibrosis	Knee OA and severe flexion contractures Ko et al. (2019)	Immobilizing knee in rats Hagiwara et al. (2008); Sasabe et al. (2017); Baranowski et al. (2019)
		Patellar Tendon Fibrosis in a Rabbit Overuse Model Liu et al. (2020)
		Binding Protein 10 losing mouse model Lim et al. (2021) ACLR-induced arthrofibrosis in rats Kaneguchi et al. (2021)

### 5.1 Frozen shoulder

Frozen Shoulder (FS) is a disorder characterized by limited active and passive shoulder movement (Zuckerman and Rokito, 2011). The pathology and stages of this disease are related to inflammation and the formation of extensive fibrotic tissue. It was found that the pathophysiological changes of FS were consistent with the obvious increase of ECM collagen, mainly COL 1a1 and COL 3a1, and the inflammatory factors were significantly increased. Previous studies had shown that fibroblasts were the most abundant cell type in the shoulder joint capsule of FS compared with normal joint capsules and played a key role in determining where inflammation occurs and promoting its persistence (Rodeo et al., 1997; Tchou et al., 2013). Once activated, fibroblasts produced TNFα, IL-1, IL-6, cyclooxygenase-2, the polysaccharide hyaluronan and inflammatory chemokines, thereby sustaining leukocyte recruitment to the inflamed synovium (Akbar et al., 2019). Furthermore, the transition from fibroblasts to myofibroblasts during frozen shoulder might be regulated by TGF-β1, and RLX-2 might reduce collagen expression by inhibiting TGF-β1 signaling in FS. Experiments have proved that continuous low-dose injection of RLX-2 into the animal shoulder joint of rats could effectively improve the range of motion of the adhesive shoulder joint, and proved That RLX-2 could effectively prevent the excessive production of myofibroblasts (Blessing et al., 2019). However, there were insufficient studies *in vitro* on the molecular mechanism of RLX in synovial fibroblasts of FS. Since frozen shoulder pathologically was involved in abnormal blood vessel proliferation (Zhao et al., 2021), we suspected that RLX might inhibit fibrosis by regulating the formation of blood vessels in the shoulder joint. Therefore, *in vivo* studies on RLX treatment for frozen shoulder were still needed. To improve the metabolic efficiency of the RLX-2, follow-up studies found that RLX-loaded Poly lactide coglycolide (PLGA) microparticles (MPs) could effectively exert the anti-fibrotic effect of RLX and significantly increased the duration of RLX activity. The study by Kirsch et al. (2022) showed that RXFP1 was also expressed in periarticular tissue of the shoulder joint of FS patients. These results suggested RLX might represented a novel therapeutic target for the treatment of frozen shoulder. However, the dose and method of RLX-2 in the treatment of human shoulder joints still need to be further research, and relevant clinical trials were also worthy of consideration. Current primary non-surgical treatments are ineffective in relieving the progression of frozen shoulder because there is no effective way to break down the accumulated collagen. RLX-2 can inhibit collagen production and promotes collagen breakdown. The potent anti-fibrotic activity of RLX-2 opens a new avenue for the treatment of frozen shoulder. Therefore, RLX-2 may represent a potential therapeutic target for the treatment of frozen shoulder.

### 5.2 Dupuytren’s disease

Pathologically, Dupuytren’s disease is characterized by fibrotic tissue hyperplasia and contracture of the palmar fascia. Finger cramps and dysfunction may occur at the end of the disease (Badalamente et al., 1983; Black and Blazar, 2011; Karbowiak et al., 2021). Histologically, the initial phase of the disease process was characterized by the thickening of small nodules composed of proliferative cells and fibroblasts (Shih and Bayat, 2010). Furthermore, increased collagen synthesis, upregulation of reactive MMPs, and downregulation of TIMPs were main changes in the molecular biology of Dupuytren’s disease (Kang et al., 2014). Currently the best treatment for this disease is surgery, including open fascial resection, closed fasciectomy, and acupuncture fasciectomy (Ruettermann et al., 2021). However, surgical treatment did not always relieve symptoms and postoperative stiffness was common, and the recurrence rate was high (Karbowiak et al., 2021). Therefore, surgeons hope to slow or stop the progression of the disease by reducing the production of ECM early in the disease. Masterson et al. (2004) found that recombinant human RLX, as an effective anti-fibrotic medium, could promote renal matrix remodeling by promoting fibroblast proliferation, reducing α-SMA expression, and collagen synthesis. Likewise, in a bleomycin-induced human lung fibrosis study, the results showed that RLX could alter the connective tissue phenotype of human lung fibroblasts, reduce the TGF-β1- induced expression of type I and type III procollagen, promote the synthesis and secretion of MMP-1 and reduce collagen deposition (Unemori et al., 1996). To investigate whether RLX could downregulate collagen synthesis and matrix metalloproteinase expression, Kang et al. (2014) found that collagen type I and type III mRNAs were significantly reduced in myofibroblasts from patients with Dupuytren’s nodes after transfection with an adenoviral RLX construct (Ad-RLX). Moreover, some studies showed that the total collagen synthesis was reduced in cultured cells exposed to Ad-RLX compared to virus and saline controls. Interestingly, as the expression of RLX gene increased, the expression levels of MMP -1 and MMP-13 were significantly decreased (Kang et al., 2014). RLX appeared to slow disease progression by inhibiting collagen formation rather than breaking down collagen in Dupuytren’s disease. Therefore, the use of RLX in Dupuytren’s disease should be initiated at an early rather than mature stage. Although experiments *in vitro* provided the possibility for the non-surgical treatment to Dupuytren’s disease, few studies had demonstrated efficacy using recombinant RLX in animal model of Dupuytren’s disease, and convincing clinical evidence was lacking.

### 5.3 Carpal tunnel syndrome

Carpal tunnel syndrome (CTS) was a series of symptoms caused by compression of the median nerve in the carpal tunnel and was the most common peripheral nerve compression disorder (Padua et al., 2016). Non-inflammatory fibrosis and thickening of subsynovial connective tissue (SSCT) were common pathological manifestations of CTS (Ettema et al., 2006). Biochemical studies had shown that human SSCT was composed of type I, III and VI collagen and proteoglycans (Ettema et al., 2004). It was found that SSCT fibrosis was associated with increased activity of TGF-β1, which could upregulate fibroblasts proliferation and activation of (Verrecchia and Mauviel, 2002; Ettema et al., 2004). In addition, TGF-β1 could also protect ECM by inhibiting the activity of MMPs and inducing the synthetic of protease inhibitors such as plasminogen activator inhibitors-1 and TIMPs (Mauviel, 2005). RLX could attenuate fibrosis by reducing collagen deposition, mainly by inhibiting myofibroblast production and modulating MMPs/TIMPs expression. Because of this property, several studies aimed to investigate the antifibrotic effect of RLX on subsynovial fibroblasts from CTS patients. Subsynovial fibroblasts from CTS patients were transformed into subsynovial myofibroblasts by TGF-β1 and then genetically modified using Ad-RLX. After RLX gene expression, compared with the control group, the expression of MMPs mRNA increased, among which the expression of MMP-1, MMP-3, MMP-8, MMP-9 were increased significantly and the expression of α-SMA, fibronectin, phospho-Smad2, TIMP-1, and TIMP-4 were decreased significantly (Kang et al., 2017). RLX appeared to not only reduce the synthesis of ECM by reducing the expression of fibronectin, α-SMA, phospho-Smad2 and other components, but also prevent the occurrence and development of carpal tunnel syndrome fibrosis by increasing the expression of MMPs and reducing TIMPs. Although RLX showed anti-fibrotic activity in CTS synovial fibroblasts, an additional study *in vivo* and clinic trials were needed to prove that RLX could improve the symptom of carpal tunnel syndrome.

### 5.4 Scleroderma

Scleroderma or systemic sclerosis was a complex chronic connective tissue disease that primarily results in thickening and hardening of the skin in addition to interstitial fibrosis of various internal organs including lungs, heart, kidneys, gastrointestinal tract, blood vessels (Denton and Black, 2000; Simms and Korn, 2002; Denton and Khanna, 2017). Under the stimulation of various cytokines and growth factors, fibroblasts differentiated into myofibroblasts, which synthesized a large amount of matrix proteins, mainly collagen, leading to scar formation and tissue thickening. Due to the complexity and heterogeneity of scleroderma, there was currently no optimal disease-modifying treatment. As early as 1958, Casten proposed the conjecture that sagging skin in patients with scleroderma might be related to the effect of RLX on collagen fibers and the idea of using RLX to treat scleroderma (Casten and Boucek, 1958). Before the discovery of recombinant human RLX, the use of RLX in the treatment of scleroderma was hampered by the low purity of RLX isolated from animals. With the development of recombinant human RLX-2 (hrRLX-2), a potent anti-fibrotic hormone, RLX-2 has been tested to improve fibrosis in systemic sclerosis (SSc). Studies of a progressive scleroderma model by knockout of the mouse RLX gene (RLX^−/−^) had shown that mice lacking the RLX gene develop age-related skin fibrosis and thickening with abundant collagen over time increased and developed at the age of 1 month. The results showed that removing the RLX gene in mice led to a gradual accumulation of collagen in the skin, leading to fibrosis and thickening of dermis, implying that RLX had a regulatory effect on excessive collagen deposition or related skin diseases characterized by fibrosis. However, hrRLX-2 treatment of RLX^−/−^ mice resulted in complete reversal of skin fibrosis when applied to the early stages of the disease, but was ineffective when applied to a more mature stage of skin scars, during the 2-week treatment period, and RLX could inhibit TGF-β-induced collagen synthesis and secretion only when continuously exposed to human dermis, whereas collagen secretion was restored after short-term exposure (Samuel et al., 2005). A study showed that serum RLX levels were higher than normal in SSc patients, suggesting a defensive response to the fibrotic process (Giordano et al., 2005). Based on these results, we propose that RLX appears to act primarily as an anti-fibrotic agent in the early stages of fibrosis, or that adherence to RLX treatment is effective in reducing collagen deposition. New drug delivery systems or alternative therapies are needed to allow RLX work longer.

Subsequently, in a clinical trial of recombinant human RLX for scleroderma, it was found that long-term continuous subcutaneous injection of recombinant human RLX significantly reduced skin thickening and severity in patients with stable and moderate-to-severe diffuse scleroderma (Seibold et al., 2000). However, in another clinical study, subcutaneous administration of recombinant human RLX for 24 weeks did not significantly improve the patient’s overall skin score, lung function, compared with placebo, and an association between relaxin and serious renal adverse events was also noted. Note that blood pressure and renal function must be closely monitored when RLX is used for conditions other than scleroderma (Khanna et al., 2009). Meanwhile, a recent study on RLX in scleroderma showed that the expression of RXFP1 on the surface of fibroblasts in patients with scleroderma was reduced, thereby reversing the anti-fibrotic effect of RLX in systemic scleroderma, and Corallo et al. (2019) found that RXFP1 was normally expressed in normal fibroblasts and RLX could counteract TGF-β1-driven upregulation of α-SMA *via* RXFP1. Therefore, restoring RXFP1 expression in fibroblasts from scleroderma patients contributes to the anti-dermal fibrotic effect of RLX. Currently there is insufficient research on the use of RLX in systemic sclerosis. This is mainly due to the lack of sufficient phase III clinical trial evidence to demonstrate efficacy of RLX in the treatment of scleroderma. This may be related to renal injury after systemic administration of RLX. Therefore, the administration method and dosage of RLX need further study. Despite many encouraging results, clinical trials of RLX in scleroderma or systemic sclerosis are lacking.

### 5.5 Arthrofibrosis

Arthrofibrosis was the accumulation of collagen in the joints, usually following trauma, surgery, inflammation, prolonged joint immobilization, or idiopathic disease (Papagelopoulos et al., 2006; Bamshad et al., 2009; Cheuy et al., 2017; Le et al., 2017). Although the causes of arthrofibrosis vary, there was a large amount of fibrotic tissue pathologically in fibrotic joint. Current treatment options for patients with arthrofibrosis were limited in scope and effectiveness. Non-surgical treatments could only be used as mild or temporary treatment to relieve symptoms. However, surgery can lead to further deterioration and complications (Usher et al., 2019). In order to clarify molecular biology of arthrofibrosis, Hagiwara et al. (2008) found that TGF-β1 produced by capsules was a representative pro-fibrosis molecule, which involved in triggering and maintaining capsule fibrosis in an immobilized knee model. On the contrary, MMPs could directly degrade collagen to alleviate fibrosis by converting cells to a proteolytic phenotype (Page-McCaw et al., 2007). Since RLX could downregulate the TGF-β1 signaling pathway and promote the expression of MMPs, Ko et al. (2019) found that Ad-RLX could treat knee osteoarthritis flexion contracture, and the results showed that after adenovirus-mediated transfer of RLX gene into synovial fibroblasts of knee joint, RLX gene could be expressed normally, and RLX could exerts anti-fibrotic effect by inhibiting collagen synthesis and promoting collagen breakdown, whereas, in addition to the increase of MMPs and TIMPs, p-Smad2/3 expression was also increased in RLX-treated synovial fibroblasts. We suspected that RLX might be involved in ECM remodeling and appeared to play a major role in collagen degradation. This study provides the basis for RLX as an alternative therapy for early joint flexion contractures. However, the introduction of adenoviruses into human knee joints is ethically controversial, so it is important to explore a new delivery method for RLX that preserves its anti-fibrotic activity. In fact, early intervention is important to prevent fibrosis. Using RLX at the surgical site during surgery or making RLX an anti-fibrotic coating for surgical implants may offer the possibility for clinical translation of RLX.

## 6 Challenges of RLX in fibrotic ligament diseases

Currently, there is insufficient research on the use of RLX for the treatment of FLDs, although many experiments *in vitro* and *in vivo* have been conducted to demonstrate the anti-fibrosis activity of RLX, whether RLX can be used in FLDs requires further research. To solve the existing obstacles and challenges, some problems are worth analyzing.

### 6.1 Lack of solid evidence from *in vivo* studies and clinical trials

Many studies had reported that synovial cells from FLDs tissue transfected RLX gene with adenovirus could reduce the collagen production and attenuate fibrosis *in vitro*. But the effect of RLX in FLDs is lacking solid confirmation *in vivo* experiments (Kang et al., 2014; Kang et al., 2017; Ko et al., 2019; Yun et al., 2019). Meanwhile, there was little convincing clinical trials confirm the effectiveness and efficiencies of RLX for FLDs (Khanna et al., 2009), partially due to the internal heterogeneity of patients with FLDs, such as age, gender, etiology, disease stage and comorbidity.

### 6.2 Pharmacokinetic issues

Fibrosis was a chronic disease requiring long-term use of RLX (Samuel et al., 2005; Blessing et al., 2019). However, the half-life of RLX is only 1.6 h, which limits its clinical application (Kirsch et al., 2022). Therefore, RLX must be administered non-intravenously, usually requiring several times a day. Given that, patients may not adhere well to the doctor’s recommendations. While these issues can be addressed by using microinfusion pumps to continuously deliver peptides into the circulatory system of treated subjects, but new routes of administration remain to be explored. For example, allowing RLX to be digested in the gastrointestinal tract, allowing RLX to be absorbed from the intestine, *etc.* In addition, topical use of RLX as an anti-fibrotic drug can prevent drug-induced systemic side effects to a certain extent.

In fact, not only the biological effects of RLX, but also the side effects caused by RLX are considered an important study. We found that during the treatment of scleroderma with RLX, some patients experienced severe renal adverse events and decreased creatinine clearance, (Khanna et al., 2009), possibly due to renal vasoconstriction after RLX was stopped. So, after long-term use of RLX, gradual discontinuation may be required. Although animal studies indicated that RLX was not significantly toxic to the kidney (Blessing et al., 2019; Kirsch et al., 2022), the biological safety of RLX should be fully considered before the clinical application.

### 6.3 Immunologic issues

The recombinant RLX-2 currently used in clinical trials was derived from human RLX gene-2, which was consistent with RLX-2 secreted by the human body. Therefore, using recombinant RLX -2 for the treatment of FLDs somewhat lessens immune rejection. However, circulating anti-RLX antibodies could be detected in approximately 20% of patients with scleroderma following long-term systemic administration of the drug (Seibold et al., 2000), although no obvious adverse drug reactions such as allergic reactions had been reported, the presence of circulating anti-RLX antibodies in these patients could lead to the unpredictable partial inactivation of the RLX, which would have a significant negative impact on their actual efficacy.

### 6.4 Interfering therapies

Due to patient variations, such as the presence of comorbidity in patients, other prior or concurrent drug treatments have to be suspended in most clinical trials with RLX, especially, when dealing with serious life-threatening diseases, drug interference should be considered seriously. Recent studies had shown that AT1R blockers, such as Sartans, which was widely used in elderly patients who also had hypertension, could eliminate the myofibroblast response to RLX, thereby reducing or negating its antifibrotic effect (Chow et al., 2019). Therefore, the interference of drugs with the reliability of clinical results is an issue to be considered.

### 6.5 Development of RLX analogs and RXFP1 agonists

The antifibrotic effect of RLX had been reported in kinds of literatures, but numerous factors had hindered clinical translation such as drug metabolic properties, manufacturing difficulties and high costs (Pini et al., 2010; Hossain et al., 2016; Blessing et al., 2019). Therefore, solving the above problems has important implications for the clinical application of RLX. CGEN25009, an RLX analogue, had been shown to inhibit TGF-β1-induced collagen deposition and enhanced MMP-2 expression, and subsequently it was proved to exert antifibrotic effects in human dermal fibroblasts. Meanwhile, CGEN25009 had a reasonable cost/yield ratio which was a significant pharmaceutical advantage over RLX (Pini et al., 2010). So CGEN25009 might be a novel and potential treatment options for FLDs. In addition to CGEN25009, B7-33, as a single-chain peptide with a minimized structural domain of human relaxin-2, was a functionally selective agonist of RXFP1. In human cardiac fibroblasts and rat renal myofibroblasts, B7-33 showed antifibrotic efficacy comparable to that of natural relaxin-2, including stimulation of pERK1/2 activity and MMP-2 levels in fibroblasts. Meanwhile, B7-33 attenuated fibrosis in animal models of different diseases, including isoproterenol (ISO)-induced cardiomyopathy and associated fibrosis mouse models, and ovalbumin (OVA)-induced chronic allergic airway disease (AAD) mouse models. Furthermore, in contrast to human relaxin-2, B7-33 did not promote prostate tumor growth *in vivo* (Hossain et al., 2016). However, CGEN25009 and B7-33 were both administered intraperitoneally in animals to exert antifibrotic effects, the development and the clinical trial of RLX analogs and RXFP1 agonists in FLDs still needed to be investigated. The use of local delivery modalities to reduce systemic exposure to drugs appears to be a feasible solution to consider in the development of RLX analogs and RXFP1 agonists.

## 7 Conclusion

Studies conducted in the past 10 years clearly shows that RLX is an endogenous secretory hormone, and if we control the drug at an appropriate dose, RLX will show effective and safe anti-fibrosis activity. At present, the research results of RLX against fibrosis are encouraging, but the cellular and molecular mechanism of RLX against FLDs is still unclear, and it is expected to become an important research topic in the field of FLDs. Although small molecule RLX analogs and RXFP1 agonists have been shown to be effective in alleviating fibrosis, new delivery methods warrant consideration and require extensive clinical trials. In conclusion, this article outlines the biological mechanism of RLX in FLD, including the key cells, cytokines and signaling pathways involved. At the same time, the challenges and prospects of using RLX in FLD are analyzed. RLX may promise to become novel treatment for FLDs, but more work is needed.
